# Structure and function of the healthy pre-adolescent pediatric gut microbiome

**DOI:** 10.1186/s40168-015-0101-x

**Published:** 2015-08-26

**Authors:** Emily B. Hollister, Kevin Riehle, Ruth Ann Luna, Erica M. Weidler, Michelle Rubio-Gonzales, Toni-Ann Mistretta, Sabeen Raza, Harsha V. Doddapaneni, Ginger A. Metcalf, Donna M. Muzny, Richard A. Gibbs, Joseph F. Petrosino, Robert J. Shulman, James Versalovic

**Affiliations:** Department of Pathology & Immunology, Baylor College of Medicine, Houston, TX USA; Department of Molecular & Human Genetics, Baylor College of Medicine, Houston, TX USA; Department of Pediatrics, Baylor College of Medicine, Houston, TX USA; Department of Molecular Virology & Microbiology, Baylor College of Medicine, Houston, TX USA; Bioinformatics Research Laboratory, Baylor College of Medicine, Houston, TX USA; Texas Children’s Microbiome Center, Department of Pathology, Texas Children’s Hospital, Houston, TX USA; Children’s Nutrition Research Center, Houston, TX USA; Pediatric Gastroenterology, Hepatology, and Nutrition, Texas Children’s Hospital, Houston, TX USA; Human Genome Sequencing Center, Baylor College of Medicine, Houston, TX USA; Alkek Center for Metagenomics and Microbiome Research, Baylor College of Medicine, Houston, TX USA

**Keywords:** Healthy gut microbiome, Pediatric, WGS, 16S rRNA

## Abstract

**Background:**

The gut microbiome influences myriad host functions, including nutrient acquisition, immune modulation, brain development, and behavior. Although human gut microbiota are recognized to change as we age, information regarding the structure and function of the gut microbiome during childhood is limited. Using 16S rRNA gene and shotgun metagenomic sequencing, we characterized the structure, function, and variation of the healthy pediatric gut microbiome in a cohort of school-aged, pre-adolescent children (ages 7–12 years). We compared the healthy pediatric gut microbiome with that of healthy adults previously recruited from the same region (Houston, TX, USA).

**Results:**

Although healthy children and adults harbored similar numbers of taxa and functional genes, their composition and functional potential differed significantly. Children were enriched in *Bifidobacterium* spp., *Faecalibacterium* spp., and members of the *Lachnospiraceae*, while adults harbored greater abundances of *Bacteroides* spp. From a functional perspective, significant differences were detected with respect to the relative abundances of genes involved in vitamin synthesis, amino acid degradation, oxidative phosphorylation, and triggering mucosal inflammation. Children’s gut communities were enriched in functions which may support ongoing development, while adult communities were enriched in functions associated with inflammation, obesity, and increased risk of adiposity.

**Conclusions:**

Previous studies suggest that the human gut microbiome is relatively stable and adult-like after the first 1 to 3 years of life. Our results suggest that the healthy pediatric gut microbiome harbors compositional and functional qualities that differ from those of healthy adults and that the gut microbiome may undergo a more prolonged development than previously suspected.

**Electronic supplementary material:**

The online version of this article (doi:10.1186/s40168-015-0101-x) contains supplementary material, which is available to authorized users.

## Background

The gastrointestinal (GI) tract is home to one of the largest, most diverse human-associated bacterial communities. More than “fellow travelers,” our gut microbiota are essential to digestion and nutrient acquisition, intestinal development and motility, and modulation of the immune system [1–3]. Further, emerging research suggests that the gut microbiome may be intimately linked to brain development and behavior [4].

The human GI microbiome is dynamic and shaped by multiple factors, including the aging process. Previously thought to be sterile until birth, the human microbiome may be seeded in utero [5, 6]. The GI microbiome changes rapidly during infancy and early childhood and may be shaped by delivery mode, diet, antibiotics, and other exposures [7, 8]. Although many of these factors continue to influence GI communities as we age, the healthy adult gut microbiome is generally considered to be stable until older age (e.g., 65–100 years), which is characterized by declines in microbiome stability and function [9–11].

Despite recognition that the gut microbiome plays important roles in development, immunity, and health outcomes later in life [12, 13], information regarding the structure and function of the gut microbiome during childhood is limited and has focused mainly on stark comparisons related to diet and/or biogeography [14, 15]. Although it has been suggested that the gut microbiome reaches a relatively stable, adult-like state after the first 1 to 3 years of life [15, 8, 16], other evidence indicates that it continues to develop into the teenage years [13, 17, 18].

It is thought that childhood may provide opportunities for microbiome interventions to promote health or prevent disease [12]. As such, it is vital to establish a baseline understanding of pediatric GI microbiome structure and function, the degree to which these vary among healthy children, and the extent to which specific microbial features are unique to childhood, as opposed to infancy, when digestive function is immature [19, 20], or adulthood, when presumed to be mature. The goals of this study were to describe gut microbial composition and functional potential in healthy, pre-adolescent children and compare them with healthy adults. Thus, we compared matched 16S rRNA gene and shotgun metagenomic profiles of healthy children from Houston, TX, and adults recruited at the Human Microbiome Project’s (HMP) [21] Houston-based clinical site.

## Results

### Pediatric and adult subject characteristics

Thirty-seven healthy children were included in our 16S-based analysis, and a subset of these (*n* = 22) were analyzed via shotgun metagenomics (WGS). Stratifying the HMP for adults who fit our inclusion criteria provided 43 and 22 subjects for our 16S- and WGS-based analyses, respectively. Subject demographics are described in Additional file 1: Table S1, and sequence accession numbers and quality metrics are described in Additional file 2: Table S2, Additional file 3: Table S3, and Additional file 4: Table S4. Our pediatric cohort included one Asian and two subjects of mixed/unknown ancestry, limiting our analysis of race to black versus white.

### 16S rRNA gene profiles

**Fig. 1:**
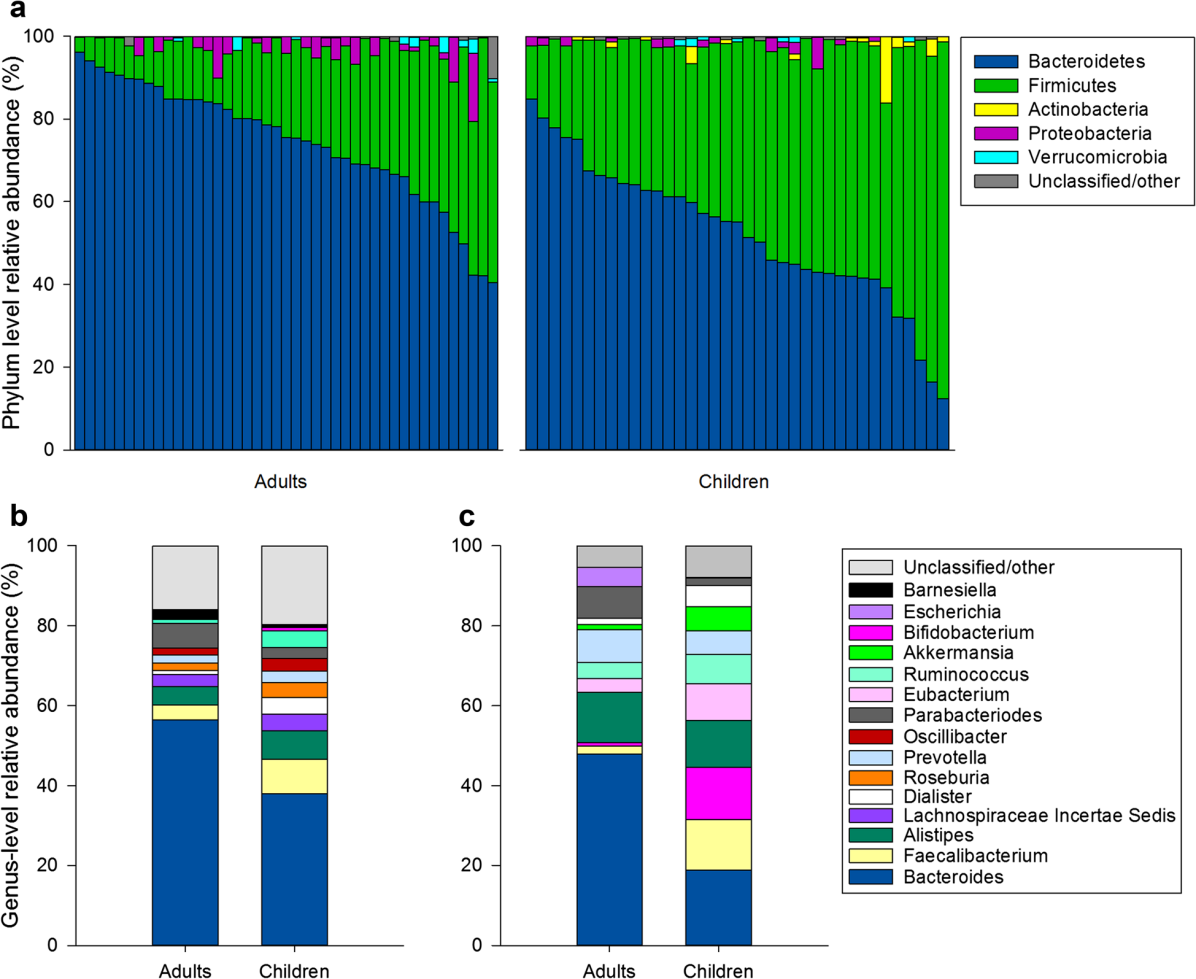
Distribution of taxa in healthy child and adult GI communities. **a** Variation in bacterial relative abundances at the phylum level via 16S rRNA gene sequencing (*n* = 37 children, 43 adults). Mean genus-level relative abundances as detected by **b** 16S sequencing (*n* = 37 children, 43 adults) and **c** shotgun metagenomic profiling (*n* = 22 children, 22 adults)

We found that, as previously reported for healthy adults, the healthy, pediatric gut microbiome is composed largely of bacteria belonging to the *Bacteroidetes* and *Firmicutes*, and the ratio of these two phyla varies considerably across subjects (Fig. 1a). In contrast to adults, the average healthy child’s gut community contains significantly lower abundances of *Bacteroidetes* and significantly greater abundances of *Firmicutes* and *Actinobacteria* (White’s non-parametric *t*-test, *q* < 0.01 for each). At the genus level, members of the *Bacteroides* accounted for nearly 40 % of the average healthy child’s gut microbiome, with *Faecalibacterium*, *Alistipes*, *Ruminococcus*, *Roseburia*, and other genera composing the balance (Fig. 1b). Although the average healthy child’s gut microbiome harbors a large variety of operational taxonomic units (OTUs), neither richness nor diversity (Additional file 5: Table S5) or overall community structure (Additional file 6: Figure S1A, C, D) varied significantly as a function of sex, ethnicity, or body mass index (BMI) status. Similarly, neither richness nor diversity differed according to race, but marginally significant differences (Adonis test, *F* = 1.59, *p* = 0.05) were detected with respect to race and variation in community structure among children (Additional file 6: Figure S1B), as well as in a combined analysis of children and adults (Additional file 7: Figure S2C).

In contrast to these results, age group (i.e., child vs. adult) appears to have a strong and significant influence on gut microbial community diversity and structure. Although the gut communities of healthy children and adults harbor similar numbers of OTUs, significant differences were detected between the two groups with respect to the Shannon and Simpson diversity indices (Mann-Whitney *U*-test, *p* < 0.05) (Additional file 8: Table S6). Despite many taxa being shared between children and adults, their distribution differed substantially. Members of the genus *Bacteroides* were the most frequently encountered taxa in both children and adults, but *Bacteroides* spp. accounted for a greater proportion of the 16S reads in adult gut communities (White’s non-parametric *t*-test, *q* < 0.01). Children’s gut communities featured significantly greater abundances of bacteria belonging to the genera *Faecalibacterium*, *Dialister*, *Roseburia*, *Ruminococcus*, and *Bifidobacterium* (White’s non-parametric *t*-test, *q* < 0.05, Fig. 1b). A full list of genera differing between children and adults is provided in Additional file 9: Table S7.

**Fig. 2:**
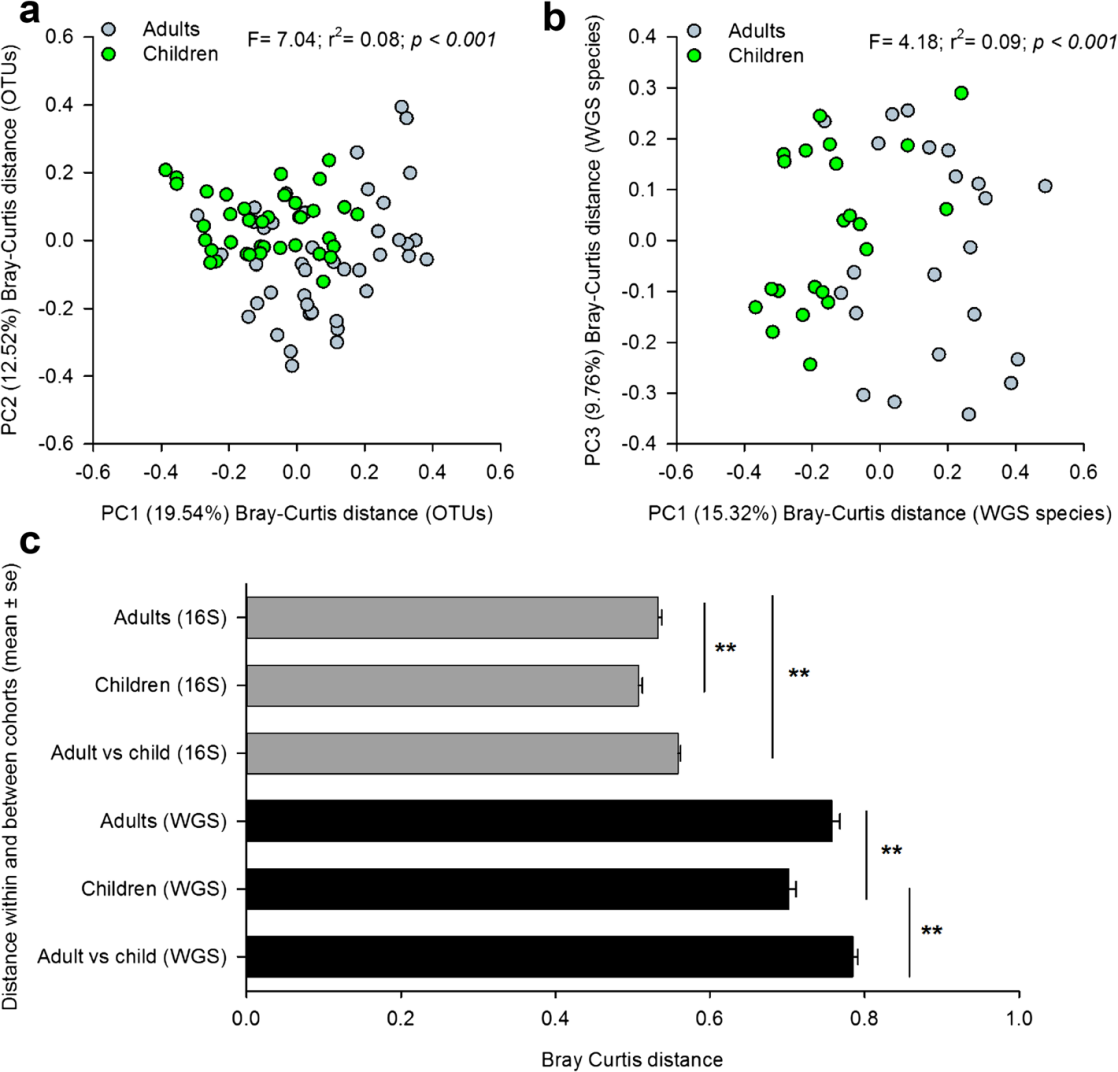
PCoA of adult and child fecal communities. Plots are based on Bray-Curtis dissimilarities of **a** 16S-based OTUs (*n* = 37 children, 43 adults) and **b** species detected via WGS (*n* = 22 children, 22 adults). The percent variation captured by each axis is indicated in *parenthesis*. Adonis test results related to age group are also presented. **c** Bray-Curtis dissimilarity within and between healthy children and adults, as a function of 16S-based OTUs or WGS-based species. **q < 0.01 by two-tailed Student’s *t*-test with 1000 permutations and Bonferroni multiple testing correction

On a global level, the gut communities of children and adults tended to be more similar to those from their respective age groups than they were to one another (Fig. 2a, c). The average within-group dissimilarities of children’s gut communities were significantly lesser than those observed among the gut communities of adults, and they are significantly lesser than those observed between adults and children (Student’s *t*-test, with 1000 permutations, *q* < 0.05, Fig. 2c). An accompanying Adonis analysis confirmed that age, either as a continuous or categorical variable, accounted for a significant proportion of the variation among subjects (continuous: *F* = 5.25, *p* < 0.001; categorical: *F* = 7.04, *p* < 0.001). The general differentiation of child and adult profiles, and the significance of age group via Adonis analysis, was consistent across distance metrics, including the weighted UniFrac (*F* = 15.47, *p* = 0.001), unweighted UniFrac (*F* = 3.25, *p* = 0.001), and the Hellinger (*F* = 5.55, *p* = 0.001) metrics (Additional file 10: Figure S3A–C). And, without exception, age accounted for a greater proportion of the variability observed among subject profiles than any other variable examined (Additional file 7: Figure S2A–D).

Random Forests analysis further validated the differentiation of child and adult gut communities. With an out-of-bag error rate of 15 % (versus a baseline error rate of 46 %), a subset of OTUs which correctly classified most subjects (68 of 80) was identified. Misclassification was not explained by known subject variables; these subjects included individuals from both sexes, all BMI classes, and multiple races, ethnicities, and subject ages. OTUs contributing to the differentiation of gut communities, according to their Random Forests importance scores, included members of the genera *Bifidobacterium*, *Faecalibacterium*, and *Bacteroides* (Table 1).

**Table 1:** Taxa contributing to the classification of child versus adult gut communities

Analysis type	Taxon identity (OTU or species)	Random forests importance score	Mean relative abundance (%)^a^		White’s non-parametric *t*-test	
			Child	Adult	*p* value	*q* value
16S	OTU_1555 *Anaerovorax*	6.77	0.23	0.16	9.99E−04	0.028
16S	OTU_1412 *Bifidobacterium*	6.07	0.34	0.01	9.99E−04	0.028
16S	OTU_1015 *Faecalibacterium*	5.28	8.26	3.52	9.99E−04	0.028
16S	OTU_411 *Collinsella*	4.79	0.20	0.01	9.99E−04	0.028
16S	OTU_2162 *Lachnospiraceae Incertae Sedis*	4.74	0.93	0.28	9.99E−04	0.028
16S	OTU_2956 *Porphyromonadaceae*	4.69	0.01	0.08	9.99E−04	0.028
16S	OTU_3384 *Bacteroides*	4.24	0.08	0.20	9.99E−04	0.028
16S	OTU_4352 *Lachnospiraceae*	4.04	3.52	1.65	1.99E−03	0.053
16S	OTU_1928 *Ruminococcaceae*	3.60	0.38	0.08	9.99E−04	0.028
16S	OTU_987 *Ruminococcaceae*	3.53	0.57	0.09	9.99E−04	0.028
WGS	*Bifidobacterium longum*	6.01	6.54	0.24	9.99E−04	0.018
WGS	*Eggerthella lenta*	5.44	0.06	0.01	1.00	1.000
WGS	*Porphyromonas asaccharolytica*	5.13	6.30E−03	0.03	4.92E−01	0.888
WGS	*Clostridium asparagiforme*	5.13	1.24E−02	6.67E−03	1.00	1.000
WGS	*Streptococcus sanguinis*	4.64	0.01	4.09E−04	1.00	1.000
WGS	*Faecalibacterium prausnitzii*	4.63	7.39	1.31	9.99E−04	0.018
WGS	*Faecalibacterium cf*	4.56	4.47	0.68	9.99E−04	0.018
WGS	*Bifidobacterium catenulatum*	4.37	0.33	0.02	8.40E−03	0.069
WGS	*Gordonibacter pamelaeae*	3.94	0.21	0.01	6.30E−02	0.313
WGS	*Granulicatella adiacens*	3.76	1.00E−03	6.36E−06	1.00	1.000

Taxa were identified as a function of their Random Forests permutation importance values. OTU identities were generated using the RDP Classifier with a confidence threshold of 50 %, and species identities were generated from the shotgun metagenomic libraries using MetaPhlAn. Differences in the relative abundance of each taxon were evaluated using two-tailed White’s non-parametric *t*-test, and Storey’s false discovery rate estimator was used to correct for multiple testing corrections within each data set (i.e., 16S, WGS)

^a^ *n* = 37 children and 43 adults in the OTU-based analysis and 22 children and 22 adults in the WGS-based analysis

### Taxonomic characterization of the metagenomic profiles

MetaPhlAn profiling of WGS libraries was used to generate genus- and species-level profiles of our healthy gut communities. Despite differences inherent between the 16S- and WGS-based approaches, we found broad agreement between the two techniques with respect to microbiome composition and its relationship with clinical variables, including age (e.g., Figs. 1b, c and 2a, b; Additional file 6: Figure S1A–H). On average, 16S and WGS libraries from the same individual shared >70 % concordance with respect to genus-level relative abundances (*n* = 44 paired specimens, average Pearson *r* = 0.71), and in many cases, these values exceeded 90 %. Similar taxa were differentially abundant between groups (Additional file 9: Table S7 and Additional file 11: Table S8), and age consistently explained a greater proportion of the variation than other known variables (Fig. 2a, b; Additional file 7: Figure S2A-H).

The number of detectable species in WGS-based profiles did not vary among children according to sex or BMI, but significant differences were observed with respect to species richness and race and ethnicity (Mann-Whitney *U*-test, *p* < 0.05, Additional file 5: Table S5). Greater numbers of species were detected in the gut communities of black versus white children and non-Hispanic versus Hispanic children. Despite differences in species richness, we did not find that ethnicity contributed significantly to the overall variation in community composition when viewed through the lens of WGS-based species profiles (Additional file 6: Figure S1E, G). The marginally significant relationship observed with respect to 16S community structure and race was recapitulated with the WGS-based species data (*F* = 1.61, *p* = 0.06; Additional file 6: Figure S1F), and marginally significant results were observed with respect to BMI (Additional file 6: Figure S1H). Despite these findings, race and BMI accounted for less variation than did age, either as a continuous (*F* = 3.56, *p* < 0.001) or categorical variable (*F* = 4.18, *p* < 0.001) (Fig. 2b). And, neither race nor BMI accounted for significant levels of variation when considered among all subjects, independent of age (Additional file 7: Figure S2F, H).

Adults and children differed significantly with respect to the number of species detected (WGS) and the diversity of those profiles (Mann-Whitney *U*-test, *p* < 0.05, Additional file 8: Table S6), with greater numbers of species and greater diversity detected in children. As observed among the 16S profiles, children’s gut communities contained a greater number of genera (Fig. 1c, Mann-Whitney *U*-test, *p* > 0.05), and the relative abundances of 13 species differed between children and adults (White’s non-parametric *t*-test, *q* < 0.10, Additional file 11: Table S8). *Faecalibacterium prausnitzii*, *Bifidobacterium longum*, and *Eubacterium rectale* were enriched in children, while *Bacteroides vulgatus* and *Bacteroides xylanisolvens* were enriched in adults.

Within- and between-group dissimilarities of WGS-based species profiles also suggest that the gut communities of children share a significantly greater degree of similarity (i.e., less dissimilarity) with one another than they do with those of adults (Student’s *t*-test with 1000 permutations, Bonferroni correction, *p* < 0.05, Fig. 2c). As with 16S, Random Forests analysis of the WGS-based species profiles correctly classified the majority of subjects by age group. With an overall error rate of 13.64 % (versus a baseline error rate of 50 %), three children and three adults were misclassified. Species contributing to the differentiation of children and adults are provided in Table 1 and include members of many of the same genera identified in our 16S analysis.

### Functional characterization of the metagenomic profiles

A total of 5820 Kyoto Encyclopedia of Genes and Genomes (KEGG) ortholog groups (KO) were detected among the 44 gut community profiles analyzed, and 46 % of these ortholog groups (2693 KO) were detected in all subjects. Gut communities of children were significantly enriched with respect to the average number of KO detected (Student’s two-tailed *t*-test, *p* = 0.02). The average healthy child’s gut metagenome contained 4446 KO, while the average adult’s contained 4201 KO.

**Fig. 3:**
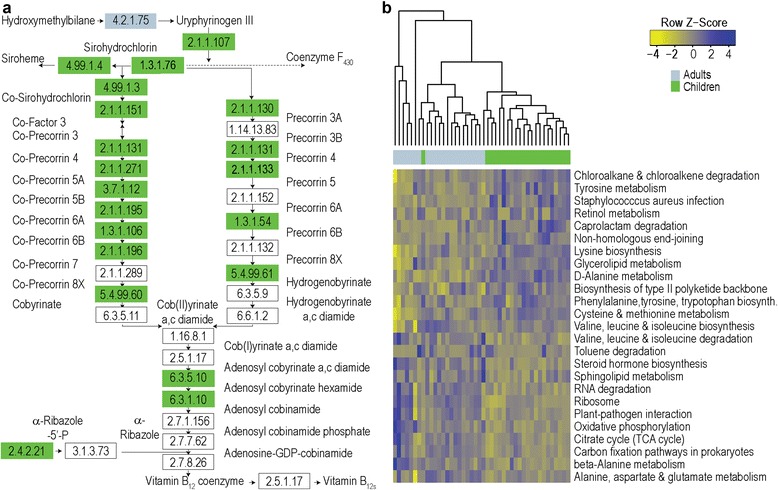
**a** The abundances of genes involved in vitamin B_12_ metabolism differed significantly between children and adults. Enrichment of KO groups (White’s non-parametric *t*-test, *q* < 0.10) is indicated by color (*green*: children; *blue*: adults). **b** Child and adult metagenomic profiles could be differentiated from one another at the pathway level. KEGG pathways with the greatest Random Forests importance scores are highlighted here

Children shared approximately 90 % similarity (median value, 10 % Bray-Curtis dissimilarity) with one another in terms of their KO profiles, a level similar to that observed among adults (median value: 87 % similarity) and slightly greater than that observed between children and adults (median value: 87 % similarity). Given such a high degree of similarity among children, no significant differences in KO abundances were detected with respect to sex, race, or ethnicity. Likewise, neither sex nor race or BMI explained a significant proportion of the variation among subject KO profiles, but marginally significant differences were detected with respect to the effects of ethnicity (*F* = 1.39, *p* = 0.07) (Additional file 6: Figure S1I–L). In contrast, the relative abundances of 1513 KO differed between healthy children and adults (White’s non-parametric *t*-test, *q* < 0.10, Additional file 12: Table S9). Notable differences included the enrichment of genes involved in vitamin B_12_ synthesis (Fig. 3a) and the de novo synthesis of folate (e.g., K03342, K11754, K02619, K03639) among children and genes involved in oxidative phosphorylation and lipopolysaccharide biosynthesis among adults.

At the pathway level, 163 KEGG pathways were detected among all profiles, and 70 % of these (114 pathways) were present in all subjects. Children shared 96 % similarity (median value, 4 % Bray-Curtis dissimilarity) with one another in terms of their KEGG pathway profiles, a level similar to that which is observed among healthy adults (median value, 95 % similarity) and between children and adults (median value, 95 % similarity). We found that neither pathway relative abundances nor overall variation with respect to functional profiles at the pathway level (Additional file 6: Figure S1M–P) differed significantly as a function of sex, race, or ethnicity among children. In contrast, we found that the relative abundances of 59 pathways differed between healthy children and adults (White’s non-parametric *t*-test, *q* < 0.10, Additional file 13: Table S10). These included pathways involved in amino acid metabolism, lipopolysaccharide biosynthesis, flagellar assembly, steroid hormone biosynthesis, RNA degradation, and oxidative phosphorylation (Fig. 3b), and many provided high discriminatory value between the profiles of children and adults in a Random Forests analysis. Our classification error rate of 17.39 % (versus a baseline error rate of 50 %) suggests that KEGG pathway profiles can be used to distinguish the gut microbiomes of most healthy children and adults from one another.

## Discussion

Although it is recognized that the gut microbiome has the potential to change along with the development of its host, information regarding the structure and function of the gut microbiome in healthy children remains limited. Previous studies have focused mainly on bacterial composition in the context of diet and/or biogeography [15, 14], and these have relied heavily on 16S data. Likewise, other adult versus pediatric comparisons have emphasized the stark contrasts of infant and adult GI communities [15, 16] rather than the more subtle contrasts of adults versus older children, have been limited to a single family [22], or have included teenagers [15, 23], despite evidence of microbiome shifts at other body sites during puberty [24, 25].

Our results suggest that the gut microbiome of healthy, pre-adolescent children is species rich and functionally complex. At the phylum level, it is dominated by *Bacteroidetes* and *Firmicutes* and, on average, harbors significantly greater abundances of *Firmicutes* and *Actinobacteria* than are generally observed in healthy adults (Fig. 1). As in adults, the pediatric gut microbiome is characterized by gradients in the abundance of *Bacteroidetes* (B) and *Firmicutes* (F) and exhibits wide variation in the B:F ratio. While the B:F ratio tends to be lower in children than in adults, both groups display substantial variability in its values, which suggests that it may not be a particularly meaningful parameter with respect to the healthy pediatric gut microbiome.

The gut communities of healthy children share 35 to 46 % similarity (54 to 65 % dissimilarity) with one another when compared taxonomically (Fig. 2c), but they share far greater similarity when compared functionally. At the ortholog group level, children share approximately 90 % similarity with one another, and at the pathway level, they share >96 % similarity. Consistent with the high degree of functional conservation observed among healthy adults [21], this suggests that there is not likely to be a single, ideal taxonomic formulation for a healthy pediatric gut microbiome. Rather, healthy pediatric gut communities may be defined by ranges of taxon abundances, combinations of which yield highly similar functional potential.

A variety of factors, including diet, sex, race, ethnicity, and obesity, are known to shape and modify the microbial communities comprising the human microbiome [14, 21, 26]. Neither we nor the HMP specifically captured dietary information from our subjects. As such, we are unable to address the effects of diet on the pediatric gut microbiome or its comparison between children and adults. Likewise, it is possible that other unknown or unrecorded factors may have influenced our findings. However, with respect to factors known to influence the human microbiome, we did not find that sex contributed significantly to variation in gut microbiome structure or function among children. This is consistent with observations among healthy adults [27], as well as in a combined analysis of adults and children (Additional file 7: Figure S2). We found that race and ethnicity had small, but statistically significant, effects on gut community richness, as well as marginally significant effects on community composition and functional gene content. As observed in the HMP [21, 27], our results suggest that race and ethnicity may contribute to variation in the gut microbiome among children. The effects of race and ethnicity were smaller than those of age group, and given the limited distribution of our study participants among racial and ethnic groups, caution in the interpretation of these results is warranted.

Data from human and animal studies suggest that gut community structure and function may be influenced by obesity status [26, 28]. Our evaluation of BMI on the pediatric gut microbiome was limited by the number of underweight, overweight, or obese children in our study (Additional file 1: Table S1). Combined analysis of adults and children failed to find that BMI accounted for a significant proportion of the variation observed with respect to 16S-based OTUs, WGS-based species composition, KO groups, or KEGG pathways (Additional file 7: Figure S2). These results mirror those reported by Finucane et al. [29] who suggest that, at scales other than the phylum level, simple signatures of obesity may not be detectable in the human microbiome.

Because the adult microbiome data we used were produced independently of our pediatric data, it is possible that technical artifacts may have influenced the results reported here. As a precaution against this, we specifically utilized identical DNA extraction methods, 16S primers, sequencing protocols, and the same sequencing center as the HMP, as each of these factors may contribute to technical bias in 16S-based studies [30]. Although these steps may not have eliminated technical bias completely, we feel that the biological signals present in the data outweigh potential artifacts, particularly given that our 16S-based results were largely and independently confirmed by WGS; our results agree broadly with previous comparisons of adult and child gut microbial communities [13, 15, 17, 22], and the functional gene-based differences we observed mirror those reported in a previous comparison of younger versus older adults [11].

Our results support and extend a growing body of evidence suggesting that GI microbial communities undergo succession in concert with the maturation and development of their human hosts. Perhaps more importantly, our results also indicate that, although the pediatric gut microbiome is characterized by levels of taxonomic and functional richness that rival those found in healthy adults, both taxonomic and functional differences distinguish the gut microbial communities of healthy children and adults from one another. Whether evaluated on the basis of 16S-based OTUs or species detected in WGS libraries, similar or significantly greater numbers of taxa were found in the gut communities of children relative to adults (Additional file 8: Table S6). Differences detected with respect to Shannon diversity index values suggest that children’s gut communities were significantly more complex than those of adults, which may reflect ongoing development. In contrast, adult communities were characterized by greater evenness (i.e., Simpson 1/D), which may reflect increased relative stability of the adult gut microbiome [9].

As reported in the study of a single family [22], we found that, on average, the gut communities of children shared a greater degree of similarity with those of other children than they did with those of adults (Fig. 2c). Likewise, the gut communities of healthy adults were more similar to those of other adults than they were to those of children. Contributing to these differences were the enrichment of *Faecalibacterium* spp. and *Bifidobacterium* spp. in children and the enrichment of *Bacteroides* spp. in adults. This particular pattern is frequently observed in studies of the human gut and aging [9, 13, 17, 31] and has also been described in the context of metabolic dysfunction and inflammation, where adults with poorer relative health tend to harbor fewer *Faecalibacterium* spp. and *Bifidobacterium* spp. and greater abundances of *Bacteroides* spp. [32] in their GI communities. Although the genus *Bacteroides* is often associated with leanness and other desirable health traits [26, 28], some of its members, including strains of *Bacteroidesfragilis*, *Bacteroides vulgatus*, and *Bacteroides dorei*, have been linked to abdominal infections, metabolic disease, and inflammation in the context of celiac disease and other GI disorders [33, 34].

**Fig. 4:**
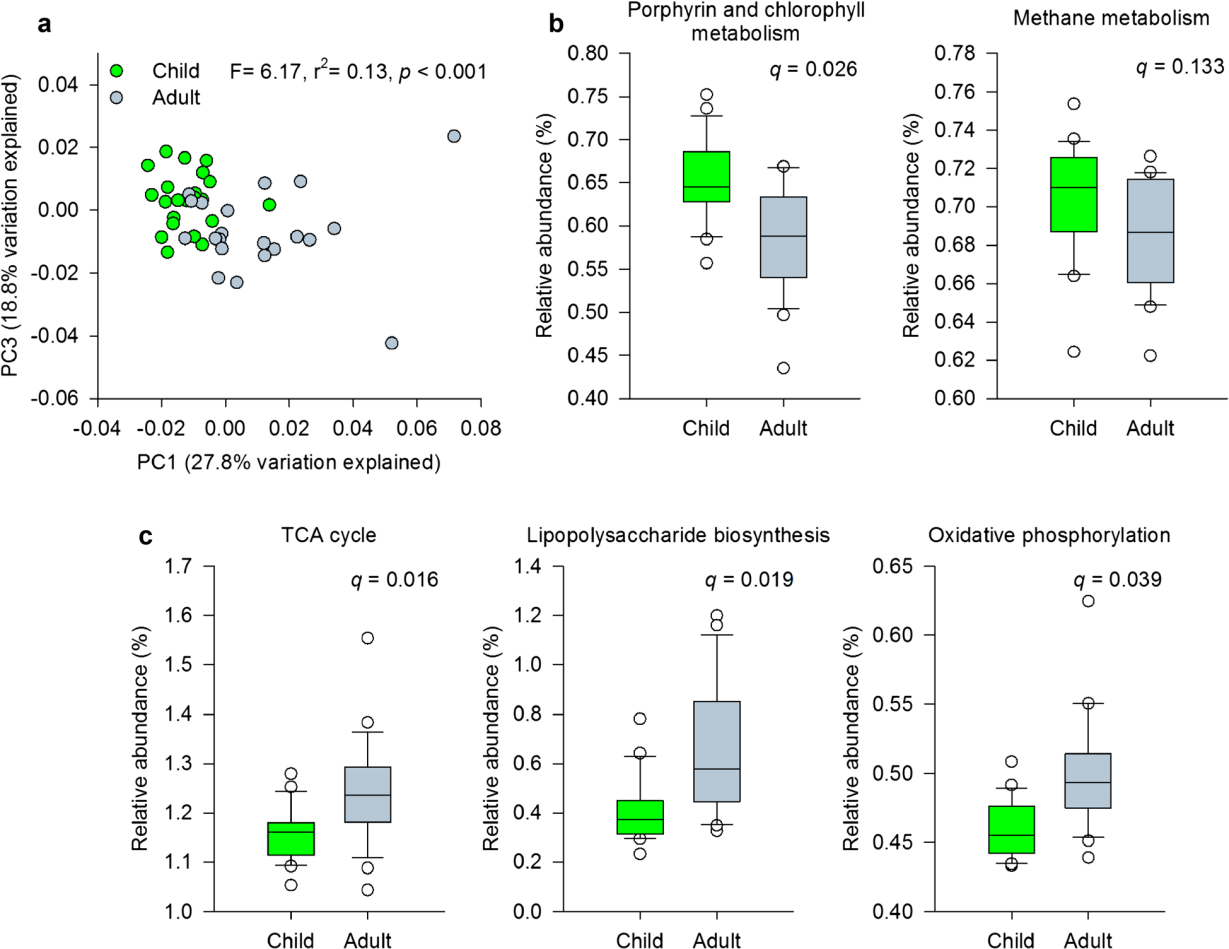
Differences in KEGG pathway profiles contribute to the differentiation of children and adults. **a** PCoA of KEGG pathway profiles from healthy children and adults (*n* = 22 children, 22 adults; Bray-Curtis dissimilarity). The percent variation captured by each axis is indicated in *parenthesis*, and an Adonis test of age group is presented. **b** KEGG pathways associated with anti-inflammatory properties were significantly enriched or trended toward enrichment in children. **c** KEGG pathways associated with pro-inflammatory processes, adiposity, and aging were significantly enriched in adults (White’s non-parametric *t*-test)

Beyond microbial composition, the gut microbiomes of healthy children also differ from those of adults in terms of functional potential. Although no single KO or pathway occurred uniquely among children or adults, we detected a small, but significant, enrichment (~6 %) in the number of gene families detected in children relative to adults. Aggregated at the pathway level, the relative abundances of approximately 25 % of KEGG pathways differed between children and adults, including some (Fig. 4b, c) previously linked to host development, metabolic syndrome, and inflammation. The gene enrichment detected among children mirrors the high versus low gene count paradigm described by Le Chatelier et al. [32], and the differential distribution of functional gene families and pathways suggests the presence of a developmental gradient with respect to microbiome functional potential and relative maturity, akin to that described in the context of healthy versus malnourished, underdeveloped children in Bangladesh [35].

In previous work describing the human microbiome and development, the gut communities of infants and adults were found to differ with respect to dietary acquisition versus de novo synthesis of nutrients by gut microbes [15]. Infant communities were significantly enriched in genes involved in the de novo synthesis of folate (vitamin B_9_), an important nutrient supporting DNA synthesis, replication, and repair [36], and the maintenance of regulatory T cells [37], while adult communities were enriched in genes directed toward dietary utilization of folate [15]. Our results suggest that by the time children reach school age, their gut communities are still significantly enriched in genes involved in folate biosynthesis, but they do not differ with respect to the abundance of genes related to dietary folate utilization.

Adult communities have also been described as having greater potential than infants [15] to produce vitamin B_12_ (cobalamin), a microbially synthesized compound with anti-inflammatory and anti-oxidant benefits and essential for neurological function [38–40]. In contrast, we found that many genes involved in cobalamin biosynthesis were significantly enriched in children (Fig. 3a). Despite the fact that vitamin B_12_ is essential at all life stages [38, 39], its concentration in the body varies with age. Although adult blood concentrations of cobalamin exceed those found in infants, cobalamin reaches its lifetime peak around 7 years of age [41]. The coordinated peaks in potential gut microbiome cobalamin production and blood cobalamin levels during childhood suggest the potential for the gut microbiome to support host development, particularly given the importance of cobalamin for neurological function [41, 40].

As with cobalamin, we also observed that the gut communities of children were enriched with respect to the metabolism of the amino acids tyrosine, lysine, cysteine, and methionine. These amino acids serve as substrates for the production of biogenic amines and neurotransmitters, both of which function as critical links along the gut-brain axis. Evidence from animal models highlights the importance of the gut microbiome in brain development, learning, and behavior [4], and although increased potential for amino acid metabolism in pediatric GI communities may play into multiple aspects of host and/or microbiome function, it may also reflect the influence of the gut microbiome on brain development and plasticity [42].

In adults, gut communities were significantly enriched with genes involved in oxidative phosphorylation, lipopolysaccharide biosynthesis, flagellar assembly, and steroid hormone biosynthesis (Fig. 3b, Additional file 13: Table S10), pathways which have been described previously in the context of inflammation. This enrichment may be a function of the gut microbiota priming the immune system [43]. It may signal increased likelihood of obesity, adiposity, and/or metabolic disease [32, 44]. Or, it may be a sign of aging, as the development of chronic, low-grade inflammation occurs both in conjunction with adiposity-related co-morbidities and as a part of the aging process [45].

Multiple gene families and functions associated with the gut microbiome affect the balance between pro- and anti-inflammatory processes. Mirroring results described by Le Chatelier et al. [32], we found that the gut microbial communities of healthy children shared traits with those from “high gene count” (i.e., healthy) individuals and were significantly enriched or showed trends toward enrichment of functions associated with anti-inflammatory properties, including vitamin B_12_ synthesis (a key component of the KEGG pathway for porphyrin and chlorophyll metabolism) and methane metabolism (Fig. 4b). In contrast, healthy adult gut communities shared many traits with “low gene count” individuals (i.e., those with low-grade inflammation and increased incidence of metabolic disorders [32]) and were significantly enriched in genes and gene families associated with inflammation and exposure to oxidative stress, including lipopolysaccharide biosynthesis, the tricarboxylic acid (TCA) cycle, and oxidative phosphorylation (Fig. 4c). These results extend previous work [11] describing the gut microbial communities of younger adults as possessing fewer pro-inflammatory traits than those of older adults and suggest that healthy children fall even lower on the pro-inflammatory scale. Further, they imply that “inflammaging” [9, 45] may not be limited to the later adult years.

## Conclusions

As with other developmental processes, childhood appears to represent a unique transitional stage with respect to the gut microbiome. Although the healthy pediatric gut microbiome harbors several adult-like features, it also retains many of its own distinct compositional and functional qualities. Such characteristics could contribute to age-adjusted definitions of the healthy gut microbiome, serve as diagnostic biomarkers to delineate life stage and direct appropriate medical treatment, and be important to consider in the development of microbiome-directed therapies, particularly those targeted toward microbiome restoration.

## Methods

### Subject recruitment and enrollment

As previously described [46], healthy children (7–12 years of age) were recruited from a large healthcare network based in Houston, TX. Informed consent was obtained from parents and assent was obtained from children. All recruitment and study procedures were approved by the Baylor College of Medicine Institutional Review Board. Exclusion criteria included, but were not limited to, abdominal pain with or without organic cause, recent major dietary changes, use of antibiotics within the prior month, probiotics within the past 6 months, and, in girls, menarche. Detailed inclusion and exclusion criteria and subject metadata are archived at dbGaP (accession phs000265.v3.p1).

### Pediatric stool collection, extraction, and sequencing

Following a research study coordinator’s instructions, participants collected stool specimens at home. Samples were stored in a sterile cup, at −20 °C, until courier transfer to the Texas Children’s Microbiome Center. Upon receipt, samples were stored at −80 °C. DNA was extracted using the PowerSoil DNA Isolation kit (MO BIO Laboratories, Carlsbad, CA, USA) with modifications to the manufacturer’s protocol [27]. DNA quality and yield were evaluated via agarose gel, Nanodrop 1000 spectrophotometer (NanoDrop, Wilmington, DE, USA), and Qubit fluorometer (Life Technologies Corporation, Carlsbad, CA, USA). Both the 16S rRNA gene and WGS libraries were generated and sequenced by the Human Genome Sequencing Center (HGSC) at Baylor College of Medicine. The 16S libraries were generated using the V3-V5 (357F/926R) primer region [46, 47]. The WGS libraries were generated using 101-bp paired-end libraries with 200-bp inserts on the HiSeq 2000 platform (Illumina Inc., San Diego, CA, USA).

### Adult microbiome data

Although the HMP recruited >240 participants, we specifically included those who were recruited at the HMP’s Houston-based clinical site and whose stool-based 16S sequence data were produced at the HGSC. This was done to limit the influence of potential sequencing-center-related biases [30, 47]. Likewise, emulation of DNA extraction and amplicon generation protocols was employed to minimize additional potential sources of bias. Forty-three HMP volunteers met the criteria described above and were included in our analysis. Stool WGS sequence data were available for 22 of the participants described above, and their WGS libraries were utilized regardless of where the data were produced. Sequence data were obtained from the NCBI Sequence Read Archive [PRJNA43017, PRJNA48479], and metadata were obtained from dbGaP [phs000228].

### Sequence analysis and community comparisons

The 16S rRNA sequence libraries were sorted by barcode and quality filtered using the Genboree Microbiome Toolset [48]. Sequences shorter than 200 bp, having average quality scores <20, including ambiguous base calls, or containing mismatches to barcode or sequencing primer were removed. After trimming barcodes and primers, all remaining reads were clustered into OTUs at a 97 % similarity threshold using QIIME (v1.3.0) [49]. OTUs were clustered using CD-Hit [50], and reads were screened for chimeras using ChimeraSlayer [51]. Potential chimeras were excluded from further analysis. OTU identities were assigned using the Ribosomal Database Project Classifier [52] with RDP training set 9 and confidence scores ≥50 %.

WGS reads were processed using a customized workflow incorporating removal of host-derived sequence, pre-assembly normalization, assembly, gene calling, and annotation steps. Bowtie2 [53] was used to map sequence reads to a reference copy of the human genome (hg19) using the “sensitive” flag. Reads with a mapped hit or mapped (paired-end) mate were removed from downstream analysis. Taxonomic profiles were generated using MetaPhlAn v1.7.7 [54], with bowtie2’s “sensitive” setting. Prior to assembly, shotgun sequence libraries were processed using digital normalization [55], a technique which removes redundant reads, reduces computational complexity, and improves assembly quality in complex metagenomes. The velvet assembler [56] (veveth, hash size 45) was used to construct contigs. Open reading frames (ORF) were identified using MetaGeneMark as implemented in MetAMOS [57]. Usearch (v5.2) [58] was used to annotate ORFs and unassembled reads with the KEGG database (v54) [59]. E-value cutoffs of 1e^−2^ and 9e^−46^ were utilized for ORFs and unassembled reads, respectively. Hits were integrated into ortholog, module, and pathway abundances using HUMAnN (v0.98) [60]. Additional details regarding the 16S rRNA gene and WGS analysis workflows are provided in Additional file 14.

Prior to calculating diversity metrics or comparing across subjects, all 16S libraries were randomly subsampled to 3700 sequences per library; the results presented here are based on subsampled data. All other taxonomic and functional data were converted to relative abundances prior to analysis. Alpha diversity metrics, including the number of species detected, the Shannon diversity index (H′), and Simpson evenness (1/D) were calculated using QIIME and compared among pediatric subgroups and between age groups. Normality was evaluated using the Shapiro-Wilk test. Student’s *t*-tests were performed, but in cases where data failed normality assumptions, Mann-Whitney *U*-tests were utilized instead. Concordance between 16S and WGS profiles was evaluated using Pearson correlations of genus-level relative abundance estimates.

Similarity among gut community profiles was evaluated with respect to sex, BMI, race, ethnicity, and/or age group using principal coordinates analysis (PCoA) of OTU data, WGS-based species abundances, KO, and KEGG pathway data. PCoA was conducted using Bray-Curtis dissimilarities, but other metrics, including weighted and unweighted UniFrac and the Hellinger distance, were also explored. Adonis tests, with 1000 permutations, were conducted in the vegan package for R (v 2.0-7) [61] to evaluate the contribution and significance of clinical variables to variation among subjects with respect to taxonomic and functional potential profiles.

Two-tailed White’s non-parametric *t*-tests [62], with Storey’s false discovery rate (FDR) corrections, were conducted in STAMP [63] and used to evaluate differences in the relative abundances of microbial taxa (including OTUs), functional gene families, and pathways with respect to age group and other subject variables. In comparisons exceeding two categories, Kruskal-Wallis H-tests were performed with Tukey-Kramer post hoc comparisons and Storey’s FDR corrections. *q* values <0.05 were considered to represent statistically significant differences, but *q* values up to 0.10 are presented for reference. Any taxon, functional gene group, or pathway which occurred in <10 % of subjects was excluded. Random Forests, a supervised learning technique which performs well with high-dimensional data and in the presence of many irrelevant features [64], was used to evaluate whether GI communities could be classified by age class and identify features differentiating children from adults. The randomForest package for R (v 4.6-7) [65] was used with the default settings. Baseline error rates were calculated as previously described [15].

The data sets supporting the results of this article are available in the NCBI Sequence Read Archive [PRJNA46339, PRJNA43017, PRJNA48479] and dbGAP [phs000228, phs000265.v3.p1].

## Additional files

Additional file 1: Table S1. Demographic and clinical features of study participants. (DOCX 16.7 kb) Additional file 2: Table S2. Subject and SRA sequence identifiers for the 16S rRNA gene data (V3-V5 region) used in this study. (XLSX 18.5 kb) Additional file 3: Table S3. Subject and SRA sequence identifiers for the shotgun metagenomic sequence data used in this study. (XLSX 14.5 kb) Additional file 4: Table S4. Sequence and assembly metrics for the shotgun metagenomic sequence libraries used in this study. (XLSX 23.0 kb) Additional file 5: Table S5. Healthy pediatric GI community richness and diversity according to 16S-based OTUs, WGS species, and subject traits. Values are presented as median with inter-quartile ranges. Within a column and clinical variable, differing letters indicate statistically significant differences (*p* < 0.05). Kruskal-Wallis H-tests with Dunn’s correction for multiple comparisons were used with BMI and race, and Mann Whitney *U*-tests were used with respect to sex and ethnicity. (DOCX 16.7 kb) Additional file 6: Figure S1. Evaluating the effects of known subject traits on pediatric GI community structure and function. PCoA of the GI microbial communities of healthy children as a function of Bray-Curtis dissimilarities and 16S-based OTUs (A–D), WGS-based species (E–H), KO groups (I–L), and KEGG pathway profiles (M–P). Variation among profiles was evaluated with respect to known traits, and the percent variation captured by each axis is indicated in parenthesis. Adonis analysis results describe the significance of each trait to overall community variation. (TIF 1.58 kb) Additional file 7: Figure S2. Evaluating the effects of known traits on community structure and function in children and adults. PCoA of the GI microbial communities of healthy children and adults as a function of Bray-Curtis dissimilarities and 16S-based OTUs (A–D), WGS-based species (E–H), KO groups (I–L), and KEGG pathway profiles (M–P). Profiles were evaluated with respect to known traits, and the percent variation explained by each axis is indicated in parenthesis. Adonis analysis results describe the significance of each trait to overall community variation. (TIF 752 kb) Additional file 8: Table S6. Healthy adult and child GI community richness and diversity according to 16S-based OTUs and WGS-based species. Values are presented as medians with inter-quartile ranges. Within a single column, a double asterisk indicates significant differences between adults and children (Mann-Whitney *U*-test, *p* < 0.05). A single asterisk indicates differences at *p* < 0.10. (DOCX 15.2 kb) Additional file 9: Table S7. Genera differing in the 16S profiles of healthy child and adult GI communities. Between-group differences were evaluated with two-tailed White’s non-parametric *t*-tests with Storey’s FDR corrections. (XLSX 14.6 kb) Additional file 10: Figure S3. Application of alternative distance metrics. PCoA of (A) unweighted UniFrac, (B) weighted UniFrac, and (C) Hellinger distances depicts the relative separation of the GI communities of healthy children and adults. The percent variation captured by each axis is indicated in parenthesis, and Adonis analysis results based on age group are provided for each metric. (TIF 3.38 mb) Additional file 11: Table S8. Species abundances which differed in the WGS-based profiles of healthy child and adult GI communities. Between-group differences were evaluated with two-tailed White’s non-parametric *t*-tests with Storey’s FDR corrections. (XLSX 11.1 kb) Additional file 12: Table S9. KEGG ortholog groups (KO) differing in the GI communities of healthy children and adults. Between-group differences were evaluated with two-tailed White’s non-parametric *t*-tests with Storey’s FDR corrections. N/A indicates KO without Enzyme Commission (EC) numbers. (TIF 155 kb) Additional file 13: Table S10. KEGG pathways differing in the functional metagenomic profiles of GI communities from healthy children and adults. Between-group differences were evaluated with White’s non-parametric *t*-tests and Storey’s false discovery rate corrections. (TIF 15.5 kb) Additional file 14:
**Supplemental methods.** (DOCX 19.5 kb)

## Abbreviations

BMI: body mass index
GI: gastrointestinal
HMP: Human Microbiome Project
KO: KEGG orthologous gene family
OTU: operational taxonomic unit
PCoA: principal coordinates analysis
RDP: Ribosomal Database Project
TCA: tricarboxylic acid
WGS: shotgun metagenomic sequencing
