# Supplementary material for: Structure and function of the healthy pre-adolescent pediatric gut microbiome
Source: Microbiome. 2015 Aug 26;3:36. doi: 10.1186/s40168-015-0101-x (PMC4550057; doi:10.1186/s40168-015-0101-x)
Supplement: Additional file 1: Table S1. — Demographic and clinical features of study participants. (DOCX 16.7 kb) [file 40168_2015_101_MOESM1_ESM.docx]

Table S1. Demographic and clinical features of study participants.

| Characteristic | 16S rRNA gene libraries | | | | Shotgun metagenomic sequence libraries | | | |
| --- | --- | --- | --- | --- | --- | --- | --- | --- |
|  | Children | | Adults (HMP) | | Children | | Adults (HMP) | |
| Subjects (count) | 37 | | 43 | | 22 | | 22 | |
| Age range (years) | 7 – 12 | | 19 – 39 | | 7 – 12 | | 19 – 32 | |
| Average age (years, mean ± SD) | 9.5 ± 1.4 | | 27 ± 4.7 | | 9.8 ± 1.4 | | 26.1 ± 3.6 | |
|  | n | % | n | % | n | % | n | % |
| Sex |  |  |  |  |  |  |  |  |
| Female/male | 19/18 | 51/49 | 21/22 | 48/52 | 12/10 | 55/45 | 11/11 | 50/50 |
| Race |  |  |  |  |  |  |  |  |
| Asian | 1 | 2.7 | 4 | 9.3 | 1 | 4.5 | 3 | 13.6 |
| Black | 10 | 27.0 | 2 | 4.6 | 7 | 31.8 | 1 | 4.5 |
| Multiracial or unknown | 2 | 5.4 | 7 | 16.3 | -- | -- | 3 | 13.6 |
| White | 24 | 64.8 | 30 | 69.7 | 14 | 63.6 | 15 | 68.2 |
| Ethnicity |  |  |  |  |  |  |  |  |
| Hispanic | 10 | 27.0 | 11 | 25.6 | 5 | 22.7 | 6 | 27.3 |
| Non-Hispanic | 27 | 73.0 | 32 | 74.4 | 17 | 77.3 | 16 | 72.7 |
| BMI status^*^ |  |  |  |  |  |  |  |  |
| Underweight BMI | 2 | 5.4 | 0 | 0 | 1 | 4.5 | 0 | 0 |
| Normal BMI | 27 | 73.0 | 25 | 58.1 | 17 | 77.3 | 14 | 63.6 |
| Overweight BMI | 6 | 16.2 | 11 | 25.6 | 1 | 4.5 | 7 | 31.8 |
| Obese BMI | 2 | 5.4 | 7 | 16.3 | 3 | 13.6 | 1 | 4.5 |

* BMI ranges are reported according CDC guidelines ([67](#_ENREF_67)):

Adults: Underweight (< 18.5), Normal (18.5 to 24.9), Overweight (25.0 to 29.9), Obese (> 30)

Children: Underweight (< 5^th^ percentile), Normal (5^th^ to less than 85^th^ percentile), Overweight (85^th^ to less than 95^th^ percentile), Obese (> 95^th^ percentile)
