# Supplementary material for: Structure and function of the healthy pre-adolescent pediatric gut microbiome
Source: Microbiome. 2015 Aug 26;3:36. doi: 10.1186/s40168-015-0101-x (PMC4550057; doi:10.1186/s40168-015-0101-x)
Supplement: Additional file 5: Table S5. — Healthy pediatric GI community richness and diversity according to 16S-based OTUs, WGS species, and subject traits. Values are presented as median with inter-quartile ranges. Within a column and clinical variable, differing letters indicate statistically significant differences (p < 0.05). Kruskal-Wallis H-tests with Dunn’s correction for multiple comparisons were used with BMI and race, and Mann Whitney U-tests were used with respect to sex and ethnicity. (DOCX 16.7 kb) [file 40168_2015_101_MOESM5_ESM.docx]

**Table S5.** Healthy pediatric GI community richness and diversity according to 16S-based OTUs, WGS-species, and subject traits. Values are presented as median with inter-quartile ranges. Within a column and clinical variable, differing letters indicate statistically significant differences (*p* < 0.05). Kruskal-Wallis H-tests with Dunn’s correction for multiple comparisons were used with BMI and race, and Mann Whitney U-tests were used with respect to sex and ethnicity.

|  | 16S rRNA gene libraries | | | | Shotgun metagenomic sequence libraries | | | |
| --- | --- | --- | --- | --- | --- | --- | --- | --- |
|  | *n* | OTUs per sample | Shannon diversity index | Simpson evenness (1/D) | *n* | Species detected | Shannon  diversity index | Simpson evenness (1/D) |
| Sex |  |  |  |  |  |  |  |  |
| Male | 18 | 164 (145 – 184) | 4.16 (3.81 – 4.67) | 1.12 (1.08 – 1.20) | 10 | 93 (79 – 99) | 3.71 (2.66 – 3.89) | 1.13 (1.11 – 1.48) |
| Female | 19 | 162 (144 – 181) | 4.04 (3.78 – 4.37) | 1.14 (1.10 – 1.20) | 12 | 92 (76 – 100) | 3.45 (3.06 – 3.66) | 1.21 (1.15 – 1.25) |
| BMI |  |  |  |  |  |  |  |  |
| Underweight | 2 | 160 (152 – 168) | 3.95 (3.53 – 4.37) | 1.17 (1.11 – 1.22) | 1 | 85 (na) | 3.51 (na) | 1.18 (na) |
| Normal | 27 | 168 (148 – 185) | 4.18 (3.78 – 4.65) | 1.13 (1.09 – 1.18) | 17 | 94 (79 – 102) | 3.50 (2.92 – 3.73) | 1.20 (1.13 – 1.34) |
| Overweight | 6 | 156 (123 – 172) | 3.85 (2.99 – 4.17) | 1.20 (1.12 – 1.40) | 1 | 95 (na) | 3.51 (na) | 1.77 (na) |
| Obese | 2 | 171 (144 – 198) | 4.23 (4.02 – 4.43) | 1.13 (1.12 – 1.14) | 3 | 73 (60 – 84) | 3.74 (3.04 – 3.92) | 1.12 (1.10 – 1.26) |
| Race |  |  |  |  |  |  |  |  |
| Asian | 1 | 173 (na^†^) | 4.95 (na) | 1.06 (na) | 1 | 95 (na) | 2.16 (na) | 1.77 (na) |
| Black | 10 | 172 (136 – 188) | 4.31 (3.98 – 4.87) | 1.11 (1.07 – 1.17) | 7 | 97 (95 – 104) ^a^ | 3.50 (2.34 – 3.85) | 1.20 (1.12 – 1.59) |
| Multi/other | 2 | 174 (162 – 187) | 4.18 (4.04 – 4.32) | 1.14 (1.14 – 1.14) | 0 | -- | -- | -- |
| White | 24 | 154 (140 – 170) | 3.98 (3.67 – 4.42) | 1.14 (1.11 – 1.20) | 14 | 82 (75 – 93) ^b^ | 3.59 (3.09 – 3.74) | 1.16 (1.13 – 1.25) |
| Ethnicity |  |  |  |  |  |  |  |  |
| Hispanic | 10 | 154 (136 – 168) | 3.96 (3.76 – 4.33) | 1.14 (1.13 – 1.20) | 5 | 73 (66 – 85) ^a^ | 3.67 (3.04 – 3.72) | 1.15 (1.13 – 1.25) |
| Non-Hispanic | 27 | 168 (150 – 185) | 4.18 (3.78 – 4.74) | 1.12 (1.08 – 1.20) | 17 | 95 (84 – 102) ^b^ | 3.50 (2.78 – 3.79) | 1.20 (1.13 – 1.44) |

^†^na, data was not sufficient to calculate the inter-quartile range
