# Supplementary figures and images for: Structure and function of the healthy pre-adolescent pediatric gut microbiome
Source: Microbiome. 2015 Aug 26;3:36. doi: 10.1186/s40168-015-0101-x (PMC4550057; doi:10.1186/s40168-015-0101-x)

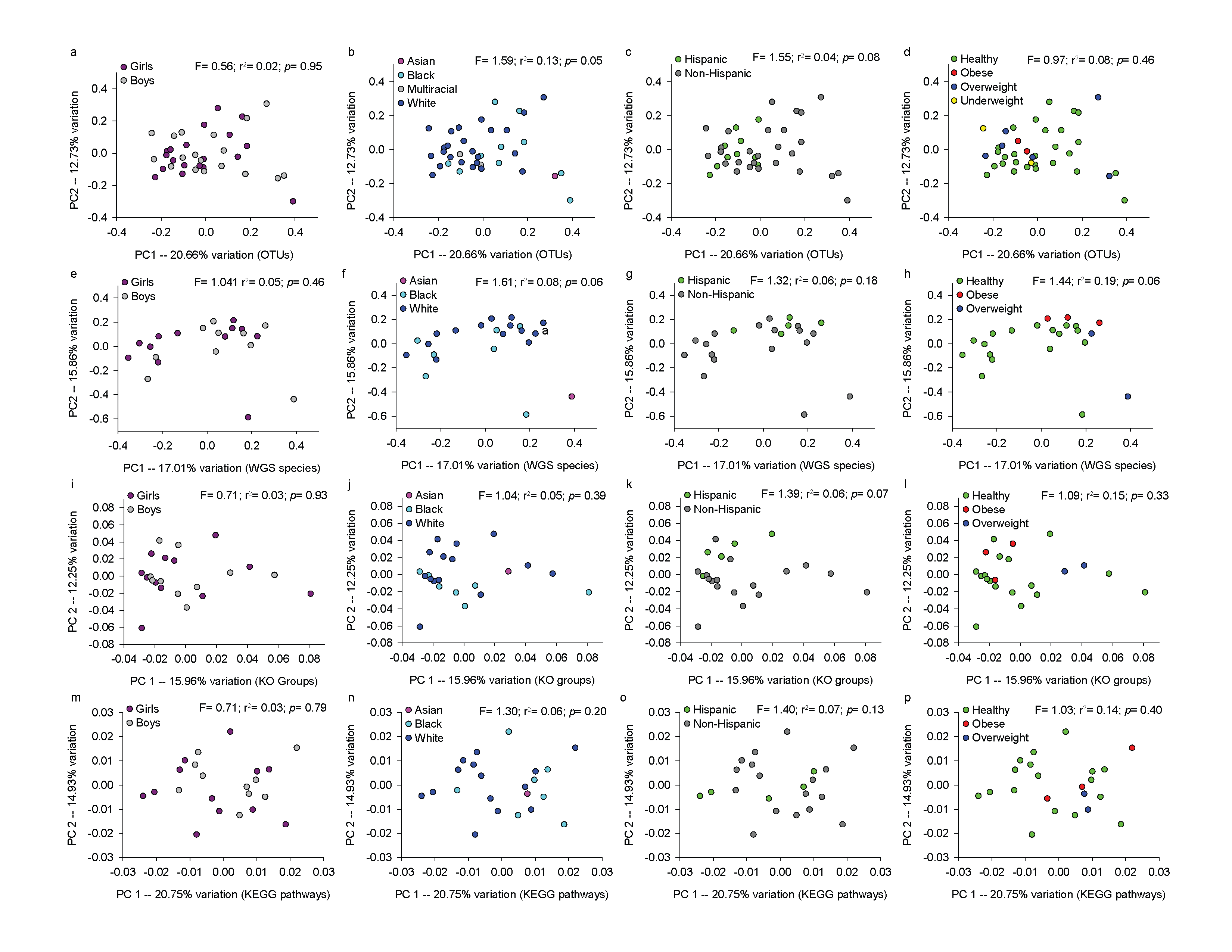

Supplement: Additional file 6: Figure S1. — Evaluating the effects of known subject traits on pediatric GI community structure and function. PCoA of the GI microbial communities of healthy children as a function of Bray-Curtis dissimilarities and 16S-based OTUs (A–D), WGS-based species (E–H), KO groups (I–L), and KEGG pathway profiles (M–P). Variation among profiles was evaluated with respect to known traits, and the percent variation captured by each axis is indicated in parenthesis. Adonis analysis results describe the significance of each trait to overall community variation. (TIF 1.58 kb) [file 40168_2015_101_MOESM6_ESM.tif]

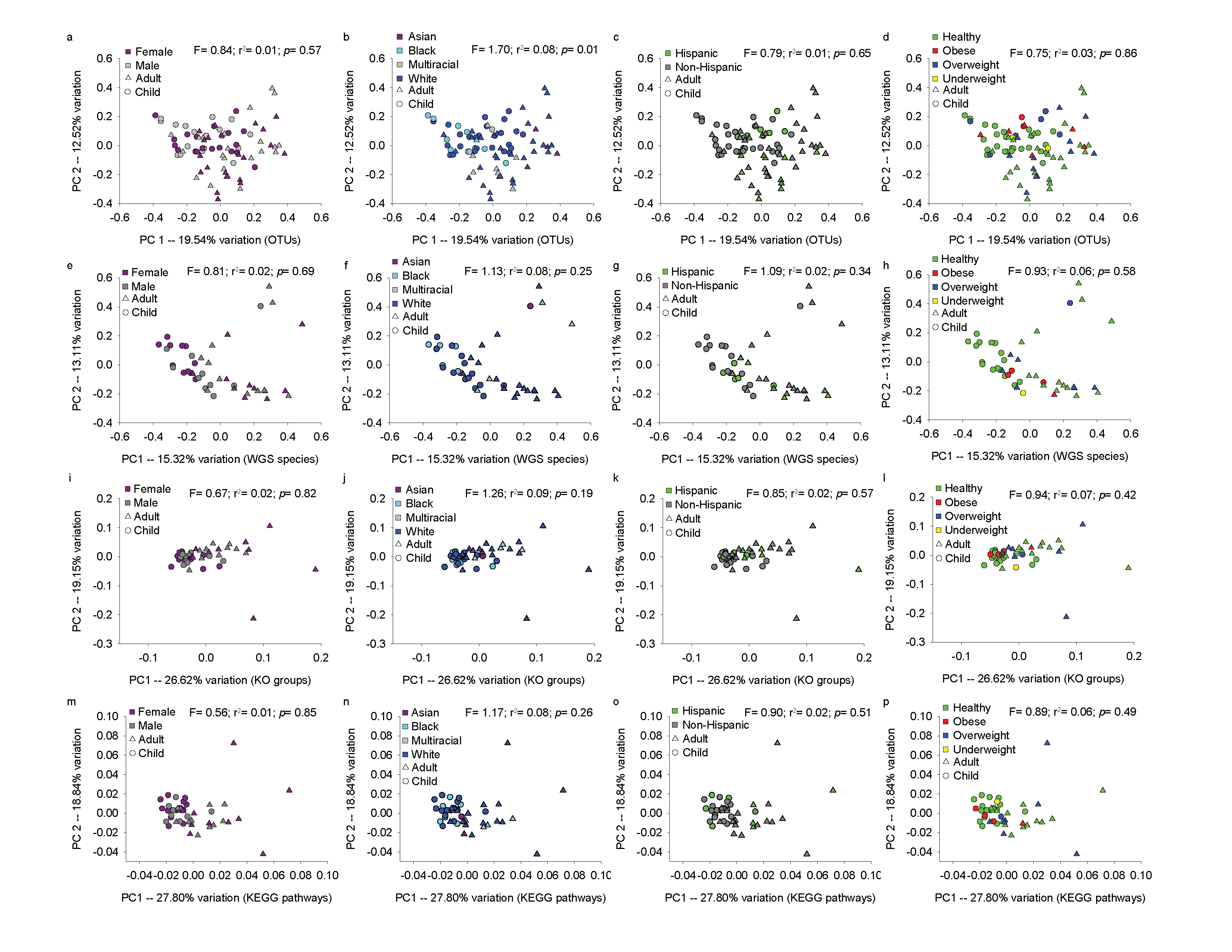

Supplement: Additional file 7: Figure S2. — Evaluating the effects of known traits on community structure and function in children and adults. PCoA of the GI microbial communities of healthy children and adults as a function of Bray-Curtis dissimilarities and 16S-based OTUs (A–D), WGS-based species (E–H), KO groups (I–L), and KEGG pathway profiles (M–P). Profiles were evaluated with respect to known traits, and the percent variation explained by each axis is indicated in parenthesis. Adonis analysis results describe the significance of each trait to overall community variation. (TIF 752 kb) [file 40168_2015_101_MOESM7_ESM.tif]
