# Supplementary material for: Structure and function of the healthy pre-adolescent pediatric gut microbiome
Source: Microbiome. 2015 Aug 26;3:36. doi: 10.1186/s40168-015-0101-x (PMC4550057; doi:10.1186/s40168-015-0101-x)
Supplement: Additional file 8: Table S6. — Healthy adult and child GI community richness and diversity according to 16S-based OTUs and WGS-based species. Values are presented as medians with inter-quartile ranges. Within a single column, a double asterisk indicates significant differences between adults and children (Mann-Whitney U-test, p < 0.05). A single asterisk indicates differences at p < 0.10. (DOCX 15.2 kb) [file 40168_2015_101_MOESM8_ESM.docx]

**Table S6.** Healthy adult and child GI community richness and diversity according to 16S-based OTUs and WGS-based species. Values are presented as medians with inter-quartile ranges. Within a single column, a double asterisk indicates significant differences between adults and children (Mann-Whitney U-test, *p* < 0.05). A single asterisk indicates differences at *p* < 0.10.

|  | 16S rRNA gene libraries^†^ | | | Shotgun metagenomic sequence libraries ^‡^ | | |
| --- | --- | --- | --- | --- | --- | --- |
|  | OTUs per sample | Shannon diversity index | Simpson evenness (1/D) | Species detected | Shannon diversity index | Simpson evenness (1/D) |
| Children | 162 (146 – 182) | 4.04 (3.78 – 4.60)^**^ | 1.14 (1.10 – 1.20)^**^ | 93 (78 – 98)^**^ | 3.50 (2.98 – 3.74) ^*^ | 1.19 (1.13 – 1.30) |
| Boys | 164 (145 – 184) | 4.16 (3.81 – 4.67) | 1.12 (1.08 – 1.20) | 93 (79 – 99) | 3.71 (2.66 – 3.89) | 1.13 (1.11 – 1.48) |
| Girls | 162 (144 – 181) | 4.04 (3.78 – 4.37) | 1.14 (1.10 – 1.20) | 93 (77 – 100) | 3.45 (3.06 – 3.66) | 1.21 (1.15 – 1.25) |
|  |  |  |  |  |  |  |
| Adults | 152 (124 – 190) | 3.62 (2.97 – 4.28)^**^ | 1.23 (1.13 – 1.40)^**^ | 62 (50 – 70)^**^ | 3.03 (2.45 – 3.55) ^*^ | 1.24 (1.16 – 1.43) |
| Men | 144 (119 – 191) | 3.43 (2.35 – 4.30) | 1.29 (1.13 – 1.52) | 64 (51 – 74) | 3.04 (2.51 – 3.67) | 1.22 (1.16 – 1.61) |
| Women | 156 (133 – 186) | 3.62 (3.21 – 4.21) | 1.21 (1.13 – 1.30) | 61 (48 – 68) | 3.01 (2.27 – 3.32) | 1.24 (1.16 – 1.41) |

^†^ *n* = 37 children and 43 adults

^‡^ *n* = 22 children and 22 adults
