# Supplementary material for: Structure and function of the healthy pre-adolescent pediatric gut microbiome
Source: Microbiome. 2015 Aug 26;3:36. doi: 10.1186/s40168-015-0101-x (PMC4550057; doi:10.1186/s40168-015-0101-x)
Supplement: Additional file 14: — Supplemental methods. (DOCX 19.5 kb) [file 40168_2015_101_MOESM14_ESM.docx]

**Supplemental methods**

As described in the main body of the text, the 16S rRNA sequence libraries were processed using the Genboree Microbiome Toolset [1], QIIME (v1.3.0) [2], and additional tools wrapped within. The specific steps, versions, and setting used in our 16S rRNA gene processing were:

1. Quality filtering was performed with the “Import 16S rRNA sequences” tool in the Genboree Microbiome Toolset, which included:
   1. removal of sequences without exact matches to their designated barcode or proximal primer sequences;
   2. trimming of barcode and primer sequences. As stated above, exact matches were required barcodes and proximal primers, while up to 3 mismatches were allowed in the detection and removal of distal primer sequences;
   3. removal of sequences containing any ambiguous base calls;
   4. removal of sequences shorter than 200 bp following barcode and primer removal;
   5. removal of sequences with average quality scores < Q20.
2. De novo clustering of quality-filtered reads using CD-Hit (v3.1.1) [3], as implemented in QIIME. Reads were clustered at a 97% similarity threshold, under default settings.
3. Selection of representative sequences performed using QIIME’s pick_rep_set.py command, which selects the most abundant read in each OTU as the representative sequence.
4. Alignment of reads was performed using the PyNAST algorithm (v1.1) [4]with the core_set_aligned.fasta.imputed file distributed with QIIME, as the alignment reference.
5. A community-wide phylogenetic tree was generated using the FastTree algorithm (v 2.1.3) [5], as implemented in the make_phylogeny.py command in QIIME.
6. Potentially chimeric sequences were flagged using ChimeraSlayer (<http://sourceforge.net/projects/microbiomeutil/files/__OLD_VERSIONS/microbiomeutil_20101212.tgz/download>) [6] and removed prior to downstream analysis.
7. Taxonomic identities were assigned to representative sequences for each OTU using the web-based implementation of the Ribosomal Database Project Classifier [7] (accessed April 28, 2014) using training set 9, with a confidence score threshold of ≥ 50%.

WGS reads were processed as follows:

1. Host-derived sequences were identified by mapping reads to a reference copy of the human genome (hg19) using Bowtie2 (v2.0.0) [8] and the “sensitive” flag. Reads with a mapped hit or mapped (paired-end) mate were removed from downstream analysis.
2. On a per-library basis, MetaPhlAn (v1.7.7) [9] was used to map sequence reads to taxonomically informative markers. Bowtie2 (v2.0.0) was used to map the paired ends via the multifastq and “sensitive” settings to the MetaPhlAn database (mpa). All profiles were later combined for downstream analysis with the merge_metaphlan_tables.py command.
3. Shotgun sequence libraries were processed using the khmer package (v1.0) [10] to remove redundant reads prior to assembly. (-C cutoff, -k k-mer size, -N number of hash tables to use, -x lower bound on hash size, --savehash saves hash for subsequent steps)
   1. normalize-by-median.py –C 20 –k 20 –N 5 –x 2.4e9 --savehash
   2. filter-abund.py --default
   3. normalize-by-median.py –C 5 –k 20 –N 5 –x 2.4e9
4. Reads were assembled into contigs on a per-sample basis using the velvet assembler (version 1.2.03) [11], specifically with the veveth algorithm and a hash size 45. Unassembled reads were retained for downstream functional annotation.
5. Open reading frames (ORF) were identified among assembled contigs using MetaGeneMark (prokaryotic version 2.8 under default settings).
6. The Usearch (v5.2) [12] was used to annotate ORFs and unassembled reads with the KEGG database (v54) [13]. E-value cutoffs of 1e^-2^ and 9e^-46^ were utilized for ORFs and unassembled reads, respectively, and a maximum of 20 hits per query were retained.
7. KEGG annotations for the ORFs and unassembled reads were merged into a single file and integrated into ortholog, module, and pathway abundances using HUMAnN (v0.98) [14] under default settings.

**Supplemental References Cited**

1. Riehle K, Coarfa C, Jackson A, Ma J, Tandon A, Paithankar S et al. The Genboree Microbiome Toolset and the analysis of 16S rRNA microbial sequences. BMC Bioinfomatics. 2012;13(Suppl 13):S11.

2. Caporaso JG, Kuczynski J, Stombaugh J, Bittinger K, Bushman FD, Costello EK et al. QIIME allows analysis of high-throughput community sequencing data. Nat Meth. 2010;7(5):335-6.

3. Li W, Godzik A. Cd-hit: a fast program for clustering and comparing large sets of protein or nucleotide sequences. Bioinformatics. 2006;22(13):1658-.

4. Caporaso JG, Bittinger K, Bushman FD, DeSantis TZ, Andersen GL, Knight R. PyNAST: a flexible tool for aligning sequences to a template alignment. Bioinformatics. 2010;26(2):266-7.

5. Price MN, Dehal PS, Arkin AP. FastTree: Computing large minimum evolution trees with profiles instead of a distance matrix. Mol Bio Evol. 2009;26(7):1641-50.

6. Haas BJ, Gevers D, Earl AM, Feldgarden M, Ward DV, Giannoukos G et al. Chimeric 16S rRNA sequence formation and detection in Sanger and 454-pyrosequenced PCR amplicons. Genome Res. 2011;21(3):494-504.

7. Wang Q, Garrity GM, Tiedje JM, Cole JR. Naive Bayesian classifier for rapid assignment of rRNA sequences into the new bacterial taxonomy. Appl Environ Microbiol. 2007;73(16):5261-7.

8. Langmead B, Salzberg SL. Fast gapped-read alignment with Bowtie 2. Nat Meth. 2012;9(4):357-9.

9. Segata N, Waldron L, Ballarini A, Narasimhan V, Jousson O, Huttenhower C. Metagenomic microbial community profiling using unique clade-specific marker genes. Nat Meth. 2012;9(8):811-4.

10. Pell J, Hintze A, Canino-Koning R, Howe A, Tiedje JM, Brown CT. Scaling metagenome sequence assembly with probabilistic de Bruijn graphs. Proc Natl Acad Sci U S A. 2012;109(33):13272-7.

11. Zerbino DR, Birney E. Velvet: algorithms for de novo short read assembly using de Bruijn graphs. Genome Res. 2008;18(5):821-9. doi:10.1101/gr.074492.107.

12. Edgar RC. Search and clustering orders of magnitude faster than BLAST. Bioinformatics. 2010;26(19):2460-1.

13. Kanehisa M, Goto S, Sato Y, Furumichi M, Tanabe M. KEGG for integration and interpretation of large-scale molecular data sets. Nucleic Acids Res. 2012;40:D109-14.

14. Abubucker S, Segata N, Goll J, Schubert AM, Izard J, Cantarel BL et al. Metabolic reconstruction for metagenomic data and its application to the human microbiome. PLoS Comput Biol. 2012;8(6):e1002358.
